# Gender but not diabetes, hypertension or smoking affects infarct evolution in ST-elevation myocardial infarction patients – data from the CHILL-MI, MITOCARE and SOCCER trials

**DOI:** 10.1186/s12872-019-1139-7

**Published:** 2019-07-03

**Authors:** David Nordlund, Henrik Engblom, Jean-Louis Bonnet, Henrik Steen Hansen, Dan Atar, David Erlinge, Ulf Ekelund, Einar Heiberg, Marcus Carlsson, Håkan Arheden

**Affiliations:** 1 Department of Clinical Physiology, Clinical Sciences, Lund University, Lund, Sweden; 2 Assistance Publique Hôpitaux de Marseille, Hôpital La Timone, Marseille, France; 3 Department of Cardiology B, Odense University Hospital, Odense, Denmark; 4 Department of Cardiology B, Oslo University Hospital Ullevål, and Faculty of Medicine, University of Oslo, Oslo, Norway; 5 Department of Cardiology, Clinical Sciences, Lund University, Lund, Sweden; 6 Department of Emergency Medicine, Clinical Sciences, Lund University, Lund, Sweden; 7 Department of Biomedical Engineering, Faculty of Engineering, Lund University, Lund, Sweden

**Keywords:** Area at risk, Gender, Sex, Diabetes, Hypertension

## Abstract

**Background:**

Infarct evolution rate and response to acute reperfusion therapy may differ between patients, which is important to consider for accurate management and treatment of patients with ST-elevation myocardial infarction (STEMI).

The aim of this study was therefore to investigate the association of infarct size and myocardial salvage with gender, smoking status, presence of diabetes or history of hypertension in a cohort of STEMI-patients.

**Methods:**

Patients (*n* = 301) with first-time STEMI from the three recent multi-center trials (CHILL-MI, MITOCARE and SOCCER) underwent cardiac magnetic resonance (CMR) imaging to determine myocardium at risk (MaR) and infarct size (IS). Myocardial salvage index (MSI) was calculated as MSI = 1-IS/MaR. Pain to balloon time, culprit vessel, trial treatments, age, TIMI grade flow and collateral flow by Rentrop grading were included as explanatory variables in the statistical model.

**Results:**

Women (*n* = 66) had significantly smaller MaR (mean difference: 5.0 ± 1.5% of left ventricle (LV), *p* < 0.01), smaller IS (mean difference: 5.1 ± 1.4% of LV, *p* = 0.03), and larger MSI (mean difference: 9.6 ± 2.8% of LV, *p* < 0.01) compared to men (*n* = 238). These differences remained significant when adjusting for other explanatory variables. There were no significant effects on MaR, IS or MSI for diabetes, hypertension or smoking.

**Conclusions:**

Female gender is associated with higher myocardial salvage and smaller infarct size suggesting a pathophysiological difference in infarct evolution between men and women.

## Introduction

Ischemic heart disease (IHD) is a major cause of death worldwide. In the acute setting of IHD, a coronary occlusion may cause ischemia which gradually develops into myocardial infarction unless the myocardium is reperfused [1–3]. Reperfusion therapy has revolutionized the care for these patients but the decision whether to reperfuse or not is sensitive to timing relative to the ischemic injury [4]. To fully utilize the potential of reperfusion therapy it’s important to understand which factors affect infarct evolution and thus may help determine when reperfusion is appropriate. In addition, the main determinant of long term prognosis in these patients have been shown to be infarct size [5–8], further stressing the need to understand the progression of the disease.

Factors such as gender, smoking, history of hypertension and diabetes have all been implicated to affect risk after acute myocardial infarction. It has been shown that women have higher mortality compared to men after acute myocardial infarction [9] although one study reported the difference only applies to younger women and might be attributed to comorbidities [10]. There are conflicting results regarding if smoking is associated with lower mortality [11] or not [12] after acute myocardial infarction, which has resulted in the concept of “smokers’ paradox” stating that smoking could be beneficial in the acute setting of myocardial ischemia. History of hypertension increases the risk for adverse events after myocardial infarction [13] and hypertension is associated with a reduced myocardial blood supply [14, 15] as well as an increased oxygen demand which may affect the pathophysiology of myocardial infarction. Presence of diabetes at the time of ischemic injury is associated with higher mortality and development of heart failure [16, 17], even when adjusted for systolic function [18].

It is not clear what mechanisms could explain differences in prognosis and pathophysiology or how they relate to the development of acute ischemic injury during coronary occlusion. Quantifying the extent of injury is therefore an important step towards better understanding of the pathophysiology associated with acute ischemic myocardial injury. Cardiovascular magnetic resonance (CMR) enables quantification of both irreversibly damaged myocardium, infarct size [19, 20], and myocardium at risk, which constitutes the myocardium that would have been injured if no reperfusion occurred [21–23] (MaR). Infarct size and MaR can be used to calculate myocardial salvage index [24] (MSI). Thus, CMR can be used to study infarct evolution [25] and which factors affect it.

Our aim was therefore to investigate to what extent gender, smoking status, presence of diabetes or history of hypertension affects the rate at which infarct evolves by assessing the amount of infarction and myocardial salvage observed using CMR in a cohort of ST elevation myocardial infarction (STEMI) patients from three recent multi-center trials.

## Methods

### Study population

Patients from the CHILL-MI (NCT01379261), MITOCARE (EudraCT Number 2010–024616-33) and SOCCER (NCT01423929) trials underwent CMR imaging 1–8 days after primary percutaneous coronary intervention (PCI) for first-time STEMI using previously published inclusion and exclusion criteria [26–29]. All patients had clinical signs of acute myocardial infarction including clinical symptoms and ECG signs consistent with STEMI, were ≥ 18 years old and had symptom duration < 6 h. Patients with a history of myocardial infarction or coronary revascularization were excluded. The CHILL-MI, MITOCARE and SOCCER trials were approved by the local or regional institutional review boards/ethics committees and all patients provided written consent.

### Trial interventions

Hypothermia was induced prior to PCI for patients in the CHILL-MI trial randomized to treatment group as previously described [26]. The SOCCER trial randomized patients to either receive oxygen therapy or room air via an OxyMask™ from inclusion in the ambulance to the end of the PCI [29]. In the MITOCARE trial, patients were randomized to receive a potentially cardioprotective compound (TRO40303) or placebo [28] at the time of acute reperfusion therapy. None of the three trial treatments showed any statistically significant effect on MaR, IS or myocardial salvage.

### Coronary angiography

Culprit vessel was determined by coronary angiography and the ischemic injury was designated as anterior (left anterior descending artery) or non-anterior (right coronary artery or left circumflex artery). Coronary artery flow before PCI was graded according to the thrombolysis in myocardial infarction (TIMI) grading system and coronary collateral flow was graded according to the Rentrop grading system [30].

### CMR

The CMR examinations were undertaken on scanners of 1.5 T field strenght from Philips (Philips Healthcare, Best, the Netherlands), Siemens (Siemens AG, Erlangen, Germany) or General Electrics (GE Healthcare, Waukesha, WI, USA). All subjects were imaged in a supine position. ECG gating was used and images were acquired at end-expiratory breath hold. Black blood triple inversion recovery T2-weighted (T2-STIR) images were acquired including full coverage of the left ventricle from base to apex prior to administration of an intravenous gadolinium-based extracellular contrast agent (0.2 mmol/kg). Contrast-enhanced steady state free precession (CE-SSFP) images were acquired approximately 5 min after contrast injection including short-axis images corresponding to the T2-STIR images. Slice thickness was 8 mm with no slice gap. In-plane resolution was typically 1.5 × 1.5 mm.

### CMR analysis

The software Segment, version 1.9R3314 (http://segment.heiberg.se), was used to analyze images [31]. MaR was quantified from the CE-SSFP images according to a previously published method [23] except for two cases where CE-SSFP images were not of diagnostic quality. In those cases, T2-STIR images were used to determine MaR since it has earlier been demonstrated that in diagnostic cases, CE-SSFP and T2-STIR images perform equally well [32]. In short, epicardial and endocardial borders of the left ventricle (LV) were delineated in T2-STIR images and in both end-diastole and end-systole for CE-SSFP images. Subsequently, MaR was identified as hyperintense myocardium and delineated in T2-STIR images and in both end-diastole and end-systole for CE-SSFP images where the mean of those two values was used. Infarct was delineated in short-axis late gadolinium enhancement (LGE) images according to a previously described automatic method where endo- and epicardium is delineated manually and a computer algorithm is applied taking partial volume effects into account [33]. Manual adjustments were performed if necessary. If present, hypointense myocardium within the hyperintense area in the CE-SSFP/T2-STIR and LGE images was included as MaR or infarct respectively (microvascular obstruction or hemorrhagic infarct).The delineations were performed by in consensus between two or out of three experienced observers (HE, MC and HA with 13, 14 and 20 years of experience) where the third observer was brought in when opinions differed between the first two. Observers had access to LGE images when delineating T2-STIR and CE-SSFP. MaR and infarct sizewere expressed as % of the LV mass while MSI was expressed as % of MaR.

### Statistical analysis

Statistical analyses were performed using SPSS (IBM, SPSS Statistics, Version 23). Continuous parameters are expressed as mean ± standard deviation and categorical parameters are expressed as per cent of total. Independent samples t-tests were used to compare population characteristics between men and women for continuous variables and Fisher’s exact test was used for categorical variables. To explain infarct development, myocardium at risk, infarct and myocardial salvage multivariable linear regressions were used. MaR and infarct were described as % of left ventricular mass while MSI was described as % of MaR. Univariable linear regression analysis was used to determine which parameters to include in the multivariable analysis. The parameters included in the univariable analysis were: age (continuous variable), pain to balloon time (continuous variable), female gender (yes/no), troponin T levels of < 15 ng/L (yes/no), left ventricular mass (LVM, continuous variable), LVM per body surface area (LVM/BSA, continuous variable), current smoker (yes/no), current smoker or ex-smoker (yes/no), diabetes (yes/no), hypertension (yes/no), treated with hypothermia (yes/no), treated with oxygen (yes/no), treated with TRO40303 (yes/no), anterior injury (yes/no), TIMI 0 flow before reperfusion (yes/no), Rentrop grade 0 of the culprit vessel (yes/no). BSA was calculated by the Du Bois Formula [34]. Multivariable linear regression analyses were performed using all parameters with *p* < 0.10 from the univariable analyses. As LVM and LVM/BSA have a high covariance they were included in separate multivariable analyses where the ones including LVM/BSA can be found in the Appendix. The variance inflation factor was found to be < 2.0 for all variables included in the multivariable analysis. A *p*-value of < 0.05 was considered to indicate statistical significance.

## Results

### Study population

**Fig. 1:**
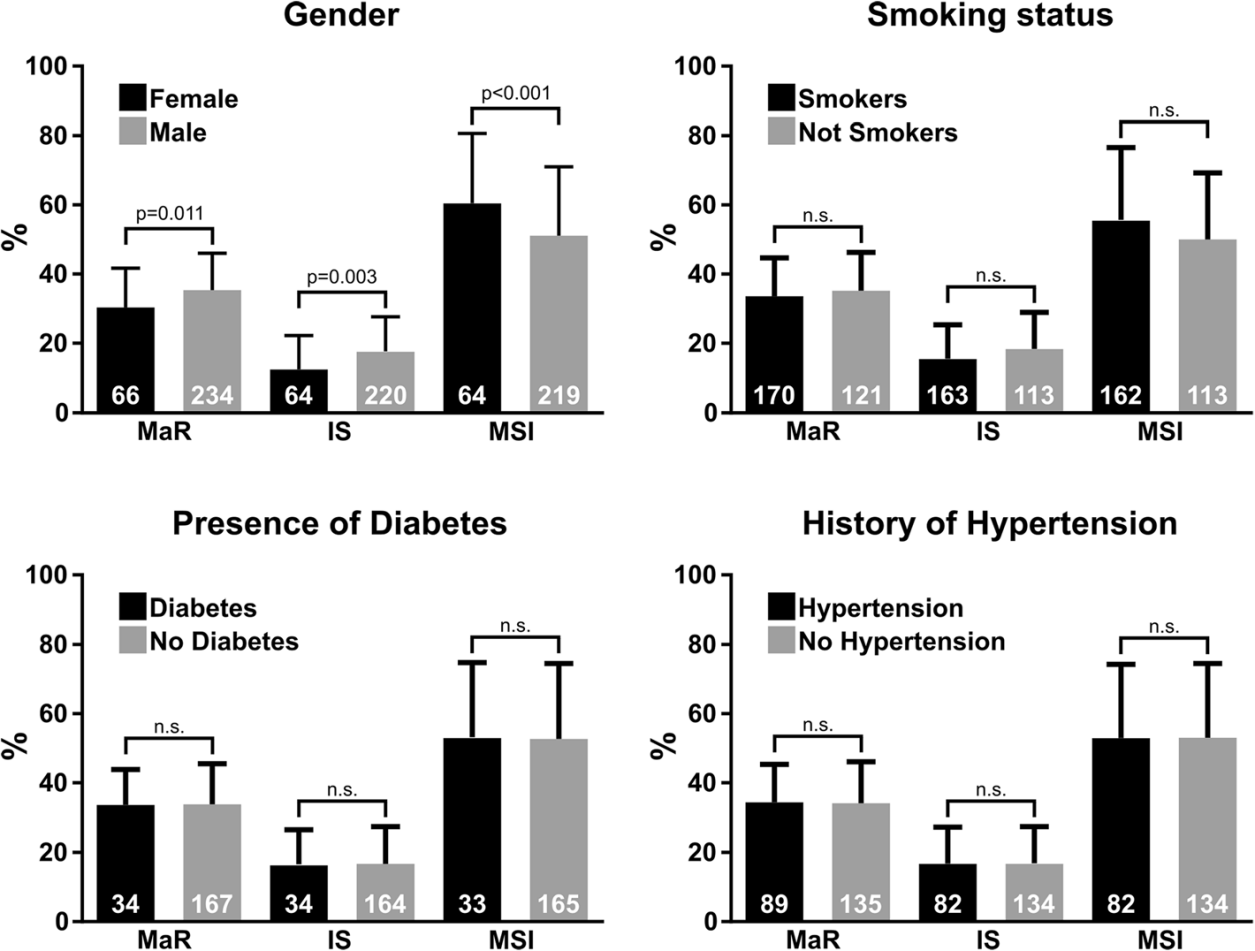
Comparisons of MaR, IS and MSI for gender, smoking status, presence of diabetes and history of hypertension. The number of datapoints included in each analysis is shown in white on the staples. The *p*-values shown are adjusted for confounders by multivariable analysis as can be seen in Tables 3, 4 and 5. MaR, IS and MSI are shown as % of left ventricular mass. MaR = Myocardium at risk, IS=Infarct size, MSI = Myocardial salvage index

**Fig. 2:**
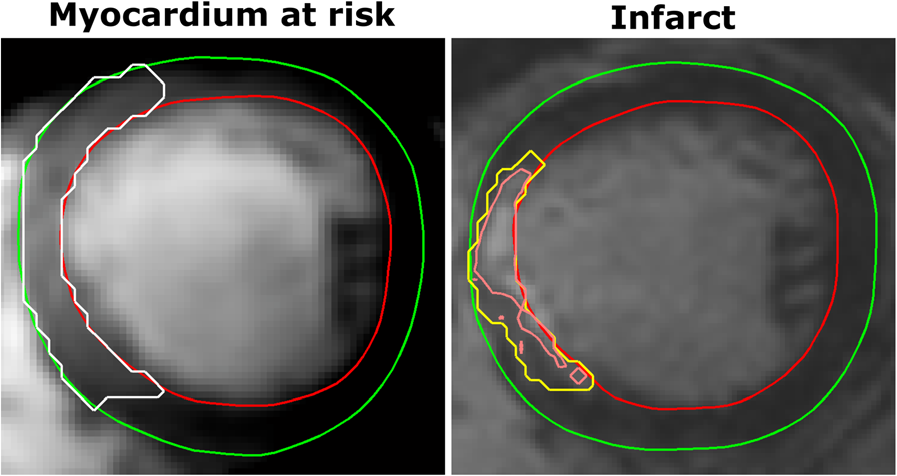
Example CMR images illustrating the measurement of myocardium at risk and infarct. The left image shows one time-frame from a mid-ventricular stack of a CE-SSFP cine stack. The green line denotes epicardium, the red line endocardium and the white line the borders of myocardium at risk. The right image shows the corresponding slice from a late gadolinium enhancement stack used to quantify infarct size using a previously published method [33]. In short, the yellow line shows the area of interest while the inner pink line takes intensities and partial volume effects into account. MaR in this patient was 23% of LVM, infarct size was 7% of LVM and the resulting myocardial salvage index was 70% of the MaR

Diagnostic CMR data of MaR was available from 298 subjects, of infarct from 285 subjects and of MSI from 282 subjects. Population characteristics are summarized in Table 1. An overview of the results is shown in Fig. 1. Example CMR images used to measure infarct and MaR are shown in Fig. 2.

**Table 1:** Population characteristics

	Total (*n* = 301)	Missing (n)	Women (*n* = 66)	Missing (n)	Men (*n* = 235)	Missing (n)	*p*-value
Age, years	61 ± 12	0	67 ± 10	0	60 ± 12	0	< 0.001
Pain to balloon, min	184 ± 73	103	194 ± 78	35	182 ± 72	68	0.376
Pre PCI TnT < 15 ng/L, %	48	5	38	3	51	2	0.089
Risk factors							
Current smoker, %	39	9	37	1	39	8	0.774
Smoker or ex-smoker, %	59	9	62	0	55	0	0.399
Diabetes, %	12	0	20	0	9	0	0.029
Hypertension, %	30	1	42	1	26	0	0.022
Treatments							
Hypothermia, %	16	0	14	0	17	0	0.576
Oxygen, %	48	206^a^	53	34	46	172	0.524
TRO40303, %	18	0	11	0	20	0	0.101
Angiography							
Anterior injury, %	42	1	37	1	43	0	0.395
TIMI 0, %	76	1	73	0	76	1	0.520
Rentrop 0, %	66	9	73	4	68	15	0.536
CMR							
LVM, g	126 ± 28	0	106 ± 30	0	132 ± 24	0	< 0.001
LVM/BSA, g/m^2^	65 ± 14	0	60 ± 18	0	66 ± 12	0	0.001
MaR, % of LVM	34 ± 11	3	30 ± 11	0	35 ± 11	3	0.001
Infarct, % of LVM	17 ± 10	16	13 ± 10	2	18 ± 10	14	< 0.001
MSI, % of MaR	53 ± 20	19	61 ± 20	2	51 ± 20	17	0.001

Population characteristics are shown as mean ± SD or valid per cent. CMR values of MaR and infarct are expressed as % of LVM while MSI is expressed as % of MaR

^a^Reliable data on whether a patient received oxygen in the acute phase of ischemia was only available for patients included in the SOCCER trial (*n* = 95). Pre PCI TnT < 15 ng/L = a blood sample acquired before coronary intervention showing a troponin T value < 15 ng/L, TRO40303 = the study treatment in the mitocare trial

*LVM* left ventricular mass, *BSA* body surface area, *MaR* myocardium at risk, *MSI* myocardial salvage index

### Myocardium at risk

Gender, anterior injury, LVM, and hypothermia were found to be variables associated with MaR in the univariable analysis (Table 2). Female gender was associated with smaller MaR while anterior injury and adjuvant hypothermia-treatment was associated with larger MaR in the multivariable analysis (Table 3).

**Table 2:** Univariable analysis

	MaR			Infarct size			MSI		
	Coeff	SE	*p*-value	Coeff	SE	*p*-value	Coeff	SE	*p*-value
Age	0.0	0.1	0.866	0.1	0.1	0.213	− 0.2	0.1	0.059
Pain to balloon	0.0	0.0	0.262	0.0	0.0	0.512	−0.0	0.0	0.143
Female gender	−5.0	1.5	0.001	−5.0	1.4	< 0.001	9.8	2.8	0.001
Pre PCI TnT < 15 ng/L	1.0	1.3	0.419	−1.8	1.2	0.148	5.8	2.4	0.015
LVM	0.1	0.0	0.004	0.1	0.0	< 0.001	−0.2	0.0	< 0.001
LVM/BSA	0.1	0.0	0.053	0.1	0.0	0.001	−0.3	0.1	0.001
Risk factors									
Current smoker	−1.0	1.3	0.465	−2.4	1.3	0.057	3.8	2.5	0.138
Smoker or ex-smoker	−1.5	1.3	0.237	−2.8	1.2	0.021	5.3	2.4	0.030
Diabetes	−0.7	2.0	0.719	−0.1	1.9	0.972	0.0	3.8	0.997
Hypertension	0.1	1.4	0.966	0.1	1.3	0.936	−0.2	2.7	0.929
Treatments									
Hypothermia	2.8	1.7	0.100	−0.2	1.6	0.882	3.0	3.2	0.357
Oxygen	2.2	2.3	0.346	−0.2	2.2	0.940	3.7	5.0	0.467
TRO40303	1.4	1.7	0.408	1.0	1.6	0.544	−1.7	3.2	0.603
Angiography									
Anterior injury	10.9	1.1	< 0.001	9.0	1.1	< 0.001	−9.4	2.4	< 0.001
TIMI 0	3.5	1.5	0.019	5.7	1.3	< 0.001	−12.4	2.7	< 0.001
Rentrop 0	−3.4	1.4	0.018	−2.0	1.4	0.139	0.4	2.7	0.894

Pre PCI TnT < 15 ng/L = a blood sample acquired before coronary intervention showing a troponin T value < 15 ng/L, TRO40303 = the study treatment in the mitocare trial

*LVM* left ventricular mass, *BSA* body surface area

**Table 3:** Multivariable analysis, MaR

Variable	Regression coefficient	SE	Partial correlation	*p*-value
Model r^2^ = 33				
Female gender	−3.5	1.4	−0.1	0.015
Anterior injury	11.5	1.1	0.5	< 0.001
Hypothermia	4.1	1.5	0.1	0.005
TIMI 0	3.2	1.3	0.1	0.014
Rentrop 0	−2.0	1.2	−0.1	0.095
LVM	−0.0	0.0	−0.0	0.707

LVM normalized to body surface area was also tested, showing no differences in significance levels, but as covariance with LVM is high it is presented in a separate analysis found in the Appendix

*LVM* left ventricular mass

### Infarct

Gender, current smoker, smoker or ex-smoker, anterior injury, age, LVM, LVM/BSA, and TIMI grade flow were variables found to be associated with infarct size in the univariable analysis Table 2). Female gender was associated with smaller infarct size while anterior injury and TIMI flow grade 0 before PCI was associated with larger infarct size in the multivariable analysis (Table 4).

**Table 4:** Multivariable analysis, Infarct size

Variable	Regression coefficient	SE	Partial correlation	*p*-value
Model r^2^ = 0.29				
Female gender	−3.2	1.4	−0.1	0.018
Current smoker	0.2	1.5	0.0	0.896
Smoker or ex-smoker	−1.9	1.4	−0.1	0.192
Anterior injury	8.4	1.1	0.4	< 0.001
TIMI 0	5.3	1.2	0.2	< 0.001
LVM	0.0	0.0	0.1	0.217

LVM normalized to body surface area was also tested, showing no differences in significance levels, but as covariance with LVM is high it is presented in a separate analysis found in the Appendix

*LVM* left ventricular mass

### Myocardial salvage

Gender, smoker or ex-smoker, anterior injury, age, LVM, LVM/BSA, and TIMI grade flow were variables found to be associated with MSI in the univariable analysis (Table 2). Female gender was associated with larger MSI while anterior injury, age, and TIMI flow grade 0 before PCI was associated with smaller MSI in the multivariable analysis (Table 5).

**Table 5:** Multivariable analysis, MSI

Variable	Regression coefficient	SE	Partial correlation	*p*-value
Model r^2^ = 0.20				
Age	−0.2	0.1	−0.1	0.064
Female gender	9.1	3.0	0.2	0.002
Smoker or ex-smoker	2.5	2.3	0.1	0.270
Anterior injury	−6.5	2.3	−0.2	0.005
TIMI 0	−12.0	2.6	−0.3	< 0.001
Pre PCI TnT < 15 ng/L	5.3	2.3	0.1	0.022
LVM	−0.1	0.0	−0.1	0.133

LVM normalized to body surface area was also tested, showing no differences in significance levels, but as covariance with LVM is high it is presented in a separate analysis found in the Appendix. Pre PCI TnT < 15 ng/L = a blood sample acquired before coronary intervention showing a troponin T value < 15 ng/L

*LVM* left ventricular mass

## Discussion

This study shows that in the highly controlled setting of three multi-center trials in STEMI patients, women had smaller myocardium at risk, smaller infarct size, and larger myocardial salvage index. Myocardium at risk, infarct, and MSI were unaffected by smoking, presence of diabetes, and history of hypertension.

### Gender difference

In previous studies women had poorer prognosis than men following STEMI [35, 36] and it has been debated whether this is due to differences in age and comorbidities or whether there is a biological difference, for example resulting in larger injuries in women. The worse prognosis seems, however, to be primarily short-term [36, 37] and may even be better for women long-term [37] which could be consistent with smaller infarct size since that has been shown to affect long-term prognosis [5–7].The data from this study does not support a larger injury in women but rather points towards that age- and comorbidity matched women have higher myocardial salvage and thus develop smaller injury. This is in line with a previous study by Canali et al. [38], also showing a greater MSI in women compared to men, but not with Eitel et al. [39] or Langhans et al. [40] who found no difference in infarct size, MaR or MSI between men and women. Langhans et al., however, included patients with both STEMI and non-STEMI which could potentially make a difference in the natural course of myocardial infarction and thus the size of the injury. Additionally, MaR measurements have been validated for STEMI but not NSTEMI and since the magnitude of the ischemia-reperfusion injury is likely different it is not clear if the MR measurements of MaR are accurate for NSTEMI. The study by Eitel et al. includes similar patients as in the present study but has women with significantly longer pain to balloon time than men and significantly more women with anterior injury which could explain part of what sets the results apart from this study, even if statistical adjustments were made. The aforementioned studies by Canali et al., Eitel et al., and Langhans et al. used CMR to measure infarct size and MaR. Mehilli [41] et al. performed a study on acute myocardial infarction patients where they measured size of injury and MaR using myocardial perfusion SPECT and found, similarly to this study, that women had smaller infarct size and greater myocardial salvage. In contrast to our study, Mehilli et al. included both STEMI and NSTEMI patients with wider inclusion criteria, patients receiving either thrombolysis or percutaneous coronary intervention (PCI) and patients with prior myocardial infarction while this study includes only first time STEMI-patients undergoing primary PCI.

In summary, previous results have been mixed with some studies showing that women have higher MSI and smaller infarcts, both in selected STEMI-populations and in less selected populations, while others show no difference. Our study adds evidence supporting higher MSI and smaller infarcts in women in a well-defined population with similar characteristics for men and women compared to previous results.

This difference has implications when designing cardioprotection trials, both regarding the importance of balancing men and women in the study arms and regarding how ratio of women in the trial might affect sample size [42].

As infarct size is one of the strongest predictors of long-term outcome and as high myocardial salvage show good treatment efficacy, it therefore seems that women would have the potential for better outcomes compared to men. Why this does not seem to be the case in bigger, less selected, materials needs to be further elucidated.

### Smokers’ paradox

The expression “smokers’ paradox” comes from the notion that smokers may have lower mortality compared to non-smokers after a myocardial infarction [11, 43]. There are however conflicting results showing that this may be a result of differences in baseline characteristics between smokers and non-smokers [12]. Similarly, results in this study show that smokers have smaller infarct size and higher MSI in the univariable analysis but that the difference disappears when adjusting for baseline characteristics. These results should be related to a recent study by Symons et al. who showed less left ventricular remodeling after STEMI in smokers compared to non-smokers which remained when adjusting for, among other parameters, infarct size and MSI [44]. Thus, it appears that there are still possible explanations for a better prognosis after STEMI in smokers compared to non-smokers but the results of this study imply that these explanations do not include lower MSI or infarct size.

### Diabetes and hypertension

Diabetic patients have been shown to have worse outcomes after STEMI [16, 17, 45, 46]. Based on the findings in the present study, this cannot be explained by differences in MSI or infarct size compared to non-diabetic patients. This points to other factors being important for a worse prognosis in diabetic patients, such as increased risk of recurring ischemic events [47], long term effects on cardiac/vessel innervation [48] or the addition of microvascular dysfunction to epicardial coronary disease. Compared to diabetes, the data on prognosis for patients with STEMI and a history of hypertension are more ambiguous as both worse and better prognosis has been shown [13, 49] and it has been suggested that the injury may develop due to different pathophysiological mechanisms [49]. The present study showed no difference in the development of infarct in patients with history vs no history of hypertension.

### Anterior injury and left ventricular mass

As expected, anterior injuries had larger MaR and infarct compared to non-anterior injuries. Note, however, that MSI was lower for anterior injuries suggesting that infarct develops faster for this group. Thus, larger MaR in the patients with anterior injuries might contribute to a faster infarct development. Van der Pals et al. [50] have shown presence of a lateral perfusion gradient within ischemic myocardium in dogs, where the edges of MaR are better perfused than the core during coronary occlusion. If this gradient exists in humans it could explain why injuries with larger MaR would have a relatively larger core area that is less perfused and therefore develops infarct faster.

At least one earlier study, on 100 patients, has reported a relation between the LVM and infarct size as measured by biomarkers which could not be seen in the current study [51]. It should be noted that the CMR-based measures in the current study are all normalized, either directly or indirectly, to LVM. A possible interpretation is therefore that while the area affected by ischemia is larger in absolute values in hearts with higher LVM, the rate at which infarct develops remains constant relative to this area.

### Limitations

The present study uses data from three different trials and pools data from both treatment and control groups which is a potential limitation. All three trials were negative however and the data was controlled for differences between treatment groups and controls. Reliable data on whether oxygen was administered or not during the acute phase of ischemia was available only for patients included in the SOCCER trial. However, as the trial was designed to detect differences in myocardial salvage, MaR, and infarct size, and did not detect any such differences, it is unlikely that this would affect the results in the present study. Data on pain to balloon time was missing in one of the studies. Pain to balloon time did not show any trend towards significance in the remaining data and it is thus unlikely that this will affect results.

## Conclusions

Female gender, but not diabetes, hypertension or smoking, was associated with smaller infarct size and higher myocardial salvage when adjusting for confounders, suggesting a pathophysiological difference in infarct evolution between men and women.

## Data Availability

The datasets used and/or analysed during the current study are available from the corresponding author on reasonable request.

## Appendix

Multivariable analyses using left ventricular mass (LVM) normalized to body surface area instead of plain LVM.

**Table 6:** Multivariable analysis, MaR

Variable	Regression coefficient	SE	Partial correlation	p-value
Model r^2^ = 33				
Female gender	−3.4	1.3	−0.1	0.011
Anterior injury	11.4	1.1	0.5	< 0.001
Hypothermia	4.0	1.5	0.1	0.006
TIMI 0	3.2	1.3	0.1	0.013
Rentrop 0	−2.1	1.2	−0.1	0.082
LVM/BSA	− 0.0	0.0	− 0.0	0.652

*LVM/BSA* left ventricular mass/body surface area

**Table 7:** Multivariable analysis, Infarct size

Variable	Regression coefficient	SE	Partial correlation	*p*-value
Model r^2^ = 0.29				
Female gender	−3.4	1.3	−0.1	0.009
Current smoker	0.4	1.5	0.0	0.795
Smoker or ex-smoker	−2.0	1.4	−0.1	0.164
Anterior injury	8.3	1.1	0.4	< 0.001
TIMI 0	5.4	1.2	0.2	< 0.001
LVM/BSA	0.1	0.0	0.1	0.112

*LVM/BSA* left ventricular mass/body surface area

**Table 8:** Multivariable analysis, MSI

Variable	Regression coefficient	SE	Partial correlation	*p*-value
Model r^2^ = 0.20				
Age	−0.2	0.1	−0.1	0.070
Female gender	9.5	2.8	0.2	0.001
Smoker or ex-smoker	2.5	2.3	0.1	0.271
Anterior injury	−6.5	2.3	−0.2	0.005
TIMI 0	−12.2	2.6	−0.3	< 0.001
Pre PCI TnT < 15 ng/L	5.2	2.3	0.1	0.027
LVM/BSA	−0.1	0.1	−0.1	0.107

*LVM/BSA* left ventricular mass/body surface area

## Abbreviations

CMR: Cardiavascular magnetic resonance
IHD: Ischemic heart disease
IS: Infarct size
MaR: Myocardium at risk
MSI: Myocardial salvage index
PCI: Percutaneous coronary intervention
STEMI: ST-elevation myocardial infarction
